# The phylogenetic position of *Myxobolus carnaticus *(Myxozoa, Myxosporea, Bivalvulida) infecting gill lamellae of *Cirrhinus mrigala *(Hamilton, 1822) based on 18S rRNA sequence analysis

**Published:** 2015-09

**Authors:** Sayani Banerjee, Avijit Patra, Harresh Adikesavalu, Anjan Mondal, Thangapalam Jawahar Abraham

**Affiliations:** Department of Aquatic Animal Health, Faculty of Fishery Sciences, West BengalUniversity of Animal and Fishery Sciences, Chakgaria, Kolkata, India

**Keywords:** *Cirrhinus mrigala*, *Myxobolus carnaticus*, gill myxoboliasis, Molecular phylogeny

## Abstract

Myxozoans are an economically important group of microscopic parasites best known for the diseases they cause in commercially important fish hosts. The classification of myxosporeans is generally based on the morphology of their myxospores. Without molecular data, it is very difficult to identify new or existing species. DNA sequence information is therefore, a prerequisite to taxonomic and phylogenic studies of myxosporeans. In the present study, a myxozoan parasite, *Myxobolus carnaticus*, infecting the gill lamellae of mrigal carp, *Cirrhinus mrigala*, was characterized by the 18S rRNA gene sequence. The DNA sequence of *M. carnaticus *clustered phylogenetically with other gill infecting *Myxobolus *spp. of freshwater clades, forming a dichotomy with closely related *M. pavlovskii *(HM991164) that infects the gill lamellae epithelium of silver carp, *Hypophthalmichthys molitrix *with 95% similarity. Evolutionary pair-wise distances among *M. carnaticus *and other species of myxosporeans indicated high genetic diversity among myxosporeans. The present study demonstrated that tissue tropism, host specificity and habitat play important roles in phylogenetic relationships among myxozoan species.

## INTRODUCTION

Myxoboliasis, caused by myxosporean parasites, is one of the most widely distributed fish diseases. Myxosporean species of the genera *Myxobolus, Thelohanellus, Henneguya, Kudoa, Myxidium, Myxosoma *and several others have been found to be the causative agents of the disease [1]. These are generally histozoic parasites of freshwater fish; nevertheless, coelozoic myxozoan parasites from marine fish are also available [2]. Heavy carp mortality associated with gill myxoboliasis has raised concern among fish farmers [3]. The current classification of myxosporeans is based on myxospore morphology, owing to the fact that their vegetative stages usually do not possess sufficient features for classification. Important characteristics are the size and shape of the myxospores and polar capsules, the number of shell valves, polar capsules and sporoplasms, the position of polar capsules on the plane of the suture and their location in the spore, the presence of surface ridges, projections and envelopes in the spore, characteristics of the polar filament, etc. Nevertheless, the use of such method makes morphological characterizations of similar myxosporeans very difficult. To resolve this issue, molecular taxonomy is implemented using the small subunit ribosomal DNA sequence. Smother et al. [4] were the first to use ssrDNA sequence analysis to study the phylogeny of Myxozoa. Since then, the inclusion of sequence information has become a necessary requirement for taxonomic and phylogenic studies of myxosporeans. In this report, the molecular characterization and phylogeny of *Myxobolus carnaticus *infecting the gill lamellae of *Cirrhinus mrigala *are presented.

## MATERIALS AND METHODS

For the purpose of the present study, the *Myxobolus *species infecting the inner base of gill lamellae of *Cirrhinus mrigala *(Hamilton, 1822), collected from Garia (Lat.22°27’59’’N; Long. 88°24’18’’E), South 24 Parganas District, West Bengal, India, during the routine survey of carp parasitic diseases in 2013, was characterized by morphometric and molecular techniques. Myxosporean identification was performed according to Lom and Arthur [5]. Details on spore collection, slide preparation, polar filament extrusion, iodinophylic vacuoles detection, staining, permanent mounting and micrometry are described in Mondal et al. [6]. The universal eukaryotic primers - ERIB1, 5´-ACC TGG TTG ATC CTG CCA G-3´ and ERIB10, 5´-CTT CCG CAG GTT CAC CTA CGG-3´ [7] were used for the amplification of the 18S rRNA gene by Eppendorf Master cycler Pro S. Molecular characterization of the *Myxobolus *species, viz., DNA extraction, PCR amplification, purification of amplicon, sequencing, electrophoresis and data analysis was done as described in Mondal et al. [6] and Abraham et al. [8]. The nucleotide sequence generated in the present study was then deposited in the NCBI GenBank database under accession number KF796620.

Phylogenetic analysis was performed on a selection of 18S rRNA gene sequences comprising the new sequence (KF796620) and 25 additional sequences from closely related species of freshwater and marine origin available in the NCBI GenBank database using the basic local alignment search tool (BLAST). Sequence alignment was performed by Multiple Sequence Comparison by Log**-**Expectation (MUSCLE) program [9] using MEGA6 software [10]. Bayesian Tree Estimation was applied to generate a phylogenetic tree using MrBayes One Model [11] in TOPALi v2 software [12]. A total of 100,000 generations were taken for the phylogenetic tree. Genetic distance analyses were conducted using the Kimura 2-parameter model [13] in MEGA6 [10]. Included codon positions were 1st + 2nd + 3rd + Noncoding. All positions containing gaps and missing data were eliminated.

## RESULTS AND DISCUSSION

**Figure 1:**
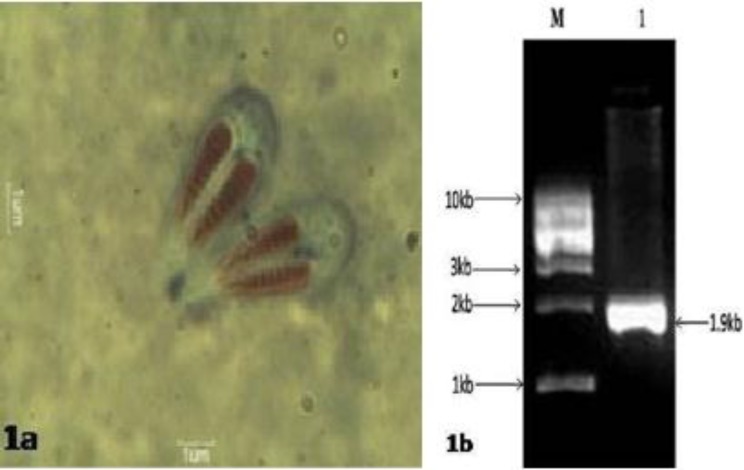
**a** **)** Wet mount preparation showing mature spores of *Myxobolus* *carnaticus* with extended polar filament at 1000X magnification (bar = 1 µm) and **(b)** Agarose gel (1.5%) showing 18S rRNA gene amplification of *Myxobolus* *carnaticus* (1984 bp) from *Cirrhinus* *mrigala*. 1: DNA ladder, 2: *Myxobolus* *carnaticus*

Myxosporean species was isolated from the inner base of the gill lamellae of sub- adult *C. mrigala*. Plasmodium was very small, white to pale coloured and elongated. Mature spores (n=20) measured 9.49±0.98 (8.10-12.90) μm in length and 8.27±0.62 (7.20-10.00) μm in breadth (Fig. 1a). Small and large polar capsules measured 2.77 (2.01-4.60) x 1.90 (1.30-3.10) µm and 3.09 (2.20-4.40) x 2.07 (1.10-3.40) μm, respectively. Polar filaments formed 9 coils inside the large polar capsule and 8 coils inside the small polar capsule. When extruded, the mean length of polar filaments ranged from 12.52 to 20.42 µm. The present species showed morphometric similarity with *Myxobolus carnaticus, *a species described by Seenappa and Manohar [14] from the inner base of hemibranchs of *C. mrigala *in Karnataka, India. In the present study, the spore length to breadth ratio (1:0.87), the large polar capsule length to breadth ratio (1:0.67) and the small polar capsule length to breadth ratio (1:0.69) differed slightly from original descriptions (1:0.79, 1:0.52 and 1:0.71, respectively) of *M. carnaticus *[14], but did not exceed the limit of natural variations typical of populations or species.

About 1984 bp fragments of the 18S rRNA gene of *M. carnaticus *were successfully amplified (Fig. 1b) by the universal eukaryotic primer sets ERIB1 and ERIB10. The novel DNA sequence of *M. carnaticus *showed 82-95% homogeneity with other myxosporeans from both freshwater and marine clades (Table 1). The 18S rDNA sequence similarity among *M. carnaticus *and other gill-infecting *Myxobolus *spp. was87-95%. Our previously characterized carp gill-infecting *M. orissae *(KF448527) [8] and *M. catmrigalae *(KC933944) exhibited 89% and 87% similarity with *M. carnaticus *(KF796620), respectively, while the carp fin-infecting *Thelohanellus caudatus *(KC865607) [6] exhibited 87% similarity with *M. carnaticus*. Maximum similarity (95%) was observed with *M. pavlovskii *(HM991164), which infects the epithelium of gill lamellae of *Hypophthalmichthys molitrix *in Hungary.

**Figure 2:**
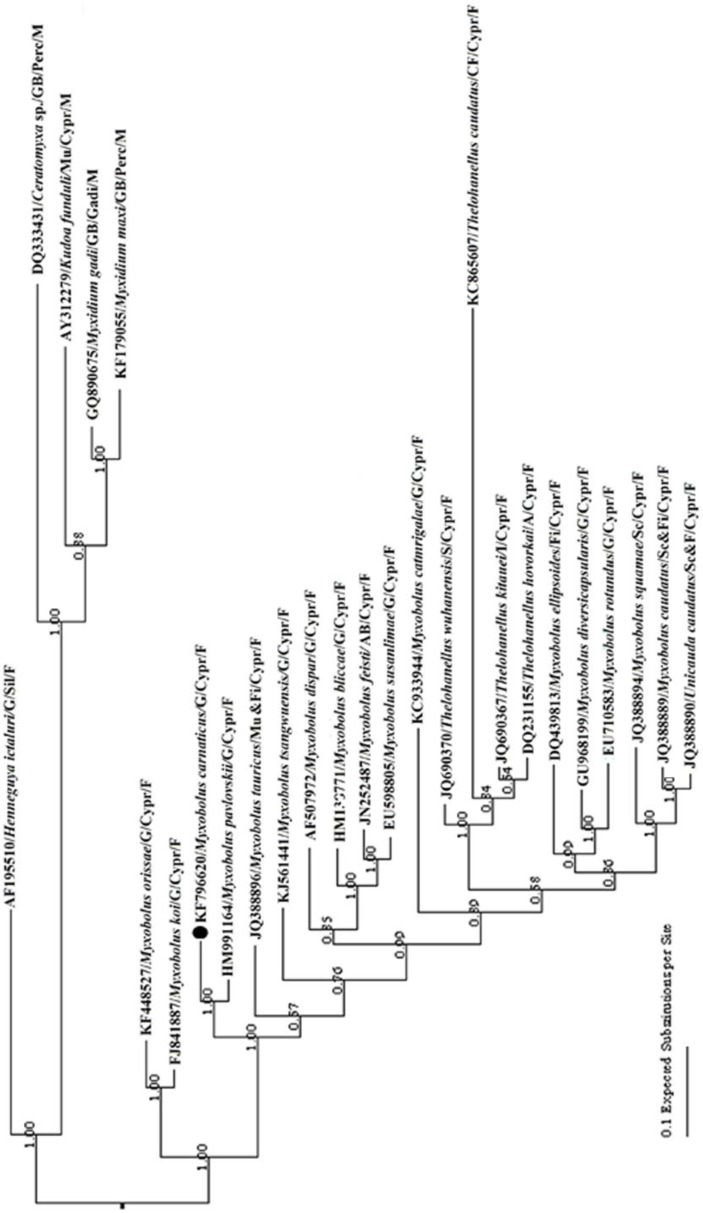
Phylogenetic tree generated by Bayesian tree estimation of the 18S rRNA gene sequences of *Myxobolus* *carnaticus* (NCBI Accession number KF796620) and other *Myxobolus* spp., and related taxa. Bootstrap confidence values are shown at nodes (100,000 replications). Abbreviations:- A: Abdomen, AB: Arteria branchialis efferens, CF: Caudal fin, Fi: Fin, G: Gill, GB: Gall bladder, I: Intestine, Mu: Muscle, Sc: Scale; Cypr: Cypriniformes, Gadi: Gadiformes, Perc: Perciformes, Sil: Siluriformes; F: Freshwater clade, M: Marine clade

The phylogenetic tree established by Bayesian Estimation for the new sequence (Fig. 2) was similar to that of Fiala [15]. The novel DNA sequence of *M. carnaticus *clustered phylogenetically with other gill infecting *Myxobolus *spp. of freshwater clades, and formed a dichotomy with carp gill infecting *M. pavlovskii *(HM991164) with a high node support. All marine species comprising of *Ceratomyxa *sp. (DQ333431), *Kudoa funduli *(AF195510), *Myxidium gadi *(GQ890675), and *Myxidium maxi *(KF179055) were phylogenetically clustered as a separate lineage (Fig. 2). *Thelohanellus *spp. clustered separately in the tree, and so did the other myxosporeans with different tissue specificity. The observed wide range in the evolutionary pair-wise distances among *M. carnaticus *and other species of myxosporeans, measured by Kimura-2 parameter algorithm (Table 1) from 0.06 (*M. pavlovskii *HM991164) to 0.30 (*Kudoa funduli *AF195510 and *Ceratomyxa *sp. DQ333431) is a possible indicator of a high level of genetic diversity among myxosporeans.

This report is the first of its kind to desribe the molecular phylogeny of *M. carnaticus *(KF796620) infecting the gill lamellae of *C. mrigala*. Earlier, we characterized *M. cuttacki *KF465682 [16] and *M. orissae *KF448527 [8] infecting carp gills and *T. caudatus *KC865607 [6] infecting caudal fins of carp from India. Myxosporeans are characterized as host, organ and tissue specific organisms [17]. According to Eszterbauer [18], site specificity is an important factor in myxozoan phylogeny.

Our study also demonstrated that tissue tropism, host specificity and habitat playimportant roles in phylogenetic relationships among myxozoan species. As the list of myxosporean parasites described from India is growing [19], molecular data on these parasites are needed to establish a genetic data-base which would help understand their taxonomy, phylogeny and genetic diversity among different ecological niches in India.

**Table 1:** Similarities of 18S rDNA sequences of *Myxobolus carnaticus* (NCBI accession number KF796620) and closely related as well as representative taxa available in NCBI GenBank database, and estimates of evolutionary divergence

	**Myxosporean ** **Species**	**Accession number**	A*	**1**	**2**	**3**	**4**	**5**	**6**	**7**	**8**	**9**	**10**	**11**	**12**	**13**	**14**	**15**	**16**	**17**	**18**	**19**	**20**	**21**	**22**	**23**	**24**	**25**	**26**
**1**	*Myxobolus carnaticus*	KF796620	100	0.00																									
**2**	*Myxobolus orissae*	KF448527	89	0.18	0.00																								
**3**	*Myxobolus koi*	FJ841887	89	0.18	0.00	0.00																							
**4**	*Myxobolus pavlovskii*	HM991164	95	0.06	0.16	0.15	0.00																						
**5**	*Myxobolus tsangwuensis*	KJ561441	90	0.08	0.15	0.15	0.05	0.00																					
**6**	*Myxobolus dispar*	AF507972	90	0.07	0.15	0.15	0.04	0.04	0.00																				
**7**	*Myxobolus feisti*	JN252487	90	0.08	0.14	0.14	0.06	0.06	0.05	0.00																			
**8**	*Myxobolus diversicapsularis*	GU968199	91	0.08	0.16	0.16	0.05	0.05	0.05	0.05	0.00																		
**9**	*Myxobolus ellipsoides*	DQ439813	92	0.08	0.16	0.16	0.05	0.06	0.05	0.05	0.03	0.00																	
**10**	*Myxobolus caudatus*	JQ388889	91	0.09	0.18	0.18	0.06	0.07	0.06	0.07	0.06	0.05	0.00																
**11**	*Myxobolus bliccae*	HM138771	90	0.08	0.15	0.15	0.05	0.05	0.04	0.04	0.04	0.05	0.06	0.00															
**12**	*Myxobolus tauricus*	JQ388896	90	0.07	0.17	0.17	0.02	0.06	0.05	0.06	0.05	0.04	0.06	0.05	0.00														
**13**	*Myxobolus susanlimae*	EU598805	90	0.07	0.15	0.15	0.05	0.05	0.04	0.02	0.04	0.04	0.06	0.03	0.05	0.00													
**14**	*Myxobolus squamae*	JQ388894	91	0.07	0.16	0.16	0.04	0.06	0.05	0.06	0.05	0.05	0.03	0.05	0.06	0.05	0.00												
**15**	*Myxobolus catmrigalae*	KC933944	87	0.13	0.21	0.21	0.11	0.11	0.11	0.13	0.11	0.13	0.12	0.12	0.12	0.12	0.11	0.00											
**16**	*Myxobolus rotundus*	EU710583	89	0.10	0.16	0.16	0.07	0.07	0.07	0.07	0.04	0.04	0.07	0.06	0.06	0.06	0.07	0.13	0.00										
**17**	*Unicauda caudatus*	JQ388890	90	0.09	0.17	0.17	0.06	0.06	0.05	0.06	0.05	0.05	0.02	0.05	0.06	0.05	0.04	0.13	0.07	0.00									
**18**	*Thelohanellus kitauei*	JQ690367	90	0.08	0.16	0.16	0.07	0.08	0.07	0.09	0.07	0.07	0.09	0.08	0.06	0.08	0.09	0.12	0.08	0.10	0.00								
**19**	*Thelohanellus caudatus*	KC865607	87	0.18	0.25	0.25	0.17	0.17	0.17	0.18	0.17	0.18	0.19	0.18	0.17	0.17	0.19	0.20	0.18	0.20	0.13	0.00							
**20**	*Thelohanellus hovorkai*	DQ231155	90	0.09	0.16	0.16	0.07	0.08	0.07	0.09	0.08	0.07	0.09	0.09	0.07	0.08	0.08	0.13	0.09	0.10	0.01	0.14	0.00						
**21**	*Thelohanellus wuhanensis*	JQ690370	89	0.08	0.16	0.16	0.07	0.08	0.06	0.09	0.08	0.07	0.08	0.08	0.07	0.07	0.08	0.13	0.09	0.09	0.02	0.14	0.02	0.00					
**22**	*Kudoa funduli*	AY312279	83	0.30	0.30	0.30	0.29	0.29	0.28	0.29	0.29	0.29	0.29	0.29	0.29	0.29	0.27	0.32	0.29	0.29	0.27	0.35	0.27	0.28	0.00				
**23**	*Henneguya ictaluri*	AF195510	84	0.20	0.17	0.17	0.19	0.19	0.18	0.17	0.18	0.18	0.19	0.18	0.19	0.17	0.19	0.24	0.19	0.19	0.18	0.28	0.18	0.18	0.29	0.00			
**24**	*Ceratomyxa *sp.	DQ333431	85	0.30	0.29	0.29	0.28	0.27	0.28	0.28	0.28	0.28	0.28	0.28	0.28	0.28	0.29	0.33	0.28	0.28	0.27	0.34	0.27	0.27	0.22	0.29	0.00		
**25**	*Myxidium gadi*	GQ890675	83	0.28	0.29	0.29	0.26	0.26	0.27	0.26	0.27	0.26	0.27	0.26	0.26	0.27	0.27	0.31	0.26	0.28	0.25	0.34	0.26	0.26	0.14	0.28	0.21	0.00	
**26**	*Myxidium maxi*	KF179055	82	0.29	0.30	0.30	0.28	0.27	0.27	0.28	0.28	0.28	0.28	0.29	0.28	0.28	0.28	0.31	0.27	0.28	0.26	0.35	0.27	0.27	0.15	0.29	0.22	0.09	0.00

## Notes

**Acknowledgments: **The work was supported by the Indian Council of Agricultural Research, Government of India, New Delhi under the Niche Area of Excellence programme. The authors thank the Vice-Chancellor, West Bengal University of Animal and Fishery Sciences, Kolkata for providing necessary infrastructure facility to carry out the work.
